# Assessment of WHO core drug use indicators at a tertiary care Institute of National importance in India

**DOI:** 10.6026/97320630018888

**Published:** 2022-10-31

**Authors:** Santenna Chenchula, Rupesh Gupta, Sunil Kumar Gupta, R Padmavathi, Saman Pathan

**Affiliations:** 1 Department of Pharmacology, AIIMS, Bhopal, India; 2 Department of Internal Medicine, Government Medical College, Shahdol, India; 3 SVS Medical College, Mahaboobnagar, Telangana, India

**Keywords:** Rational use of medicines, internal medicine, essential medicines, prescription practices, generic name, WHO core prescribing indicators

## Abstract

Rational prescribing of medicines is an important aspect of drug prescribing which helps in safe and efficacious and cost-effective drug treatment for patients. WHO Prescription indicators are intended to evaluate the services provided to the population concerning the rational use of medicines. The study aims to study prescription practices and rational use of medicines in the department of Internal medicine, using WHO prescribing indicators in a tertiary care teaching institute of national importance. A total of 50 prescriptions were digitally photographed and analysed for prescription practices and rational drug use, using standard WHO core prescribing indicators. A total of 301 drugs with multiple and diverse diagnoses were used. Statistical analysis was done using SPSS 22 version. The average number of drugs per prescription was 3.48%. It was found that only 13.79% of prescriptions have generic names, whereas 27.58% of patient encounters had at least one drug from the National List of Essential Medicine, 6.8% of prescriptions have antibiotics and 0.7% of prescriptions were injections. The number of prescriptions with fixed drug combinations was 27.55%. Indicators such as percentage of the National List of Essential Medicine, fixed drug combinations and prescribing with a generic name are used. Hence, we will suggest regular prescription audit practices and conducting CMEs and training workshops for clinicians for the rational use of medicines in all healthcare settings to succeed in the rational use of medicine.

## Background:

A drug prescription pattern audit is an important aspect of patient care, which is a part of the clinical audit which serves as a measure of the quality of care provided to the patient and helps in the improvement of patient care by changing or implementing the needed changes [1]. This is also an integral part of medical education which helps clinicians in improving prescription quality and ultimately better patient care. Many recent research studies recommended constant evaluation of the quality of prescribing patterns. Prescription error is an unacceptable medication error that is very common in many hospitals worldwide. Prescription pattern audit studies are highly useful tools in assessing the prescribing pattern and dispensing of medicines prevalent in a particular area. The main aim of these studies is to facilitate the rational use of medicines [2]. Currently several reasons such as an increase in new drug marketing, wide variations in the pattern of prescription and consumption of drugs, growing concern about delayed adverse effects, and cost of drugs all enhance the importance of prescribing patterns audit [3]. Currently, rational use of medicines is an important requirement due for many reasons, among them; antimicrobial resistance is one of the important concerns. Irrational prescribing or overuse of medicines is an arising major problem worldwide. According to World Health Organization (WHO), more than half of all medicines prescribed, are dispensed inappropriately. Overuse, under use or misuse of medicines, will lead to drug resistance, cost of treatment and duration of treatment increases which ultimately leads to wastage of resources and widespread health hazards. WHO defines the rational use of medicines (RUM) as "Patients should receive medications appropriate to their clinical needs, in doses that meet their requirements, for an adequate period, and at the lowest cost to them and their community [4]. Presently, the WHO and the National Health Policy of India, have focused on prescribing drugs by generic names from the list of essential medicines, because prescribing with the generic name is also one of the major issues to fix in India and many other countries [5-6]. This type of study is imperative to bridge the areas such as rational use of drugs, pharmaco economics, antimicrobial stewardship and evidence-based medicine.

The WHO developed core medication use indicators consisting of prescription indicators intended with an aim to assess the services provided to the population concerning medications [7]. These are universally useful for any setting in the world in any nation which is highly standardized and are recommended for inclusion in any drug usage study using these indicators. Accordingly, drug use indicators provide a simple tool for quickly and reliably assessing a few critical aspects of pharmaceutical use in primary health care (8). Results with these indicators point towards the particular drug use issues that need examination in more detail [8].WHO core prescription indicators allow for assessing the therapeutic actions taken in similar institutions, enabling subsequent comparison of parameters between them, and to evaluate the population's medication needs and determining the most commonly used medications in a given locality, to identify the prescription profile and quality of services offered to the population by the hospital. This study was designed to study the drug prescribing pattern at the medical outpatient department (OP) at our tertiary care centre which is a teaching medical college cum hospital, by using the following prescription indicators [8]:

 The WHO prescribing indicators include:

[1] The average number of drugs per prescription.

[2] Percentage of drugs prescribed by generic name

[3] Percentage of prescriptions containing antimicrobial agents (antibiotics)

[4] Percentage of injections per prescription

[5] Percentage of drugs prescribed from the EML.

Evaluation of all the prescribing indicators irrespective of the diagnosis in a particular department like in our study would enable capturing a wider picture of the current trends rather than evaluating only some particular group of drugs like anti-epileptics, antimicrobials, anti asthmatics and anti-hypertensive drugs [5]. Therefore, it is of interest to evaluate the rational use of medicines in the department of Internal medicine, at our tertiary care teaching institute of national importance depending on the WHO prescribing indicators.

## Methodology:

The present cross-sectional, OP-based study was carried out in a tertiary care teaching hospital which is an institute of national importance, in Central India, after taking ethical clearance from the institutional human ethics committee. The study was carried out for one month as a pilot study at the All India Institute of Medical Sciences, Bhopal. A total of 66 outpatient prescriptions of the internal medicine department were digitally photographed at the pharmacy of the hospital, out of which 16 prescriptions were incomplete. Prescriptions of patients attending Internal Medicine OPD and treated on an outpatient basis for their ailments were included irrespective of the comorbidities. Data were collected on the demographic details of age, gender, diagnosis, and the treatment prescribed which were mentioned in the prescription. All the prescriptions were analysed based on the following parameters:

[1] Demographic parameters (Initials, age, sex, OPD registration number, date of consultation, and legible handwriting)

[2] Medical components (History, examination, presumptive/definite diagnosis, investigations, correct dose and dosage, duration of treatment, follow-up advice, referral details, do's and don'ts, legible signature, and medical council registration number).

The prescriptions collected were assessed based on the WHO prescribing indicators

[1] The average number of drugs per prescription: Average, calculated by dividing the total number of different drug products prescribed, by the number of encounters surveyed. Irrespective of whether the patient received the drugs or not.

[2] Percentage of drugs prescribed by generic name: Percentage, calculated by dividing the number of drugs prescribed by generic name divided by the total number of drugs prescribed, multiplied by 100.

[3] Percentage of prescriptions containing antimicrobial agents (antibiotics): The percentage was calculated by dividing the number of patient encounters during which an antibiotic was prescribed, by the total number of encounters surveyed and expressed as a percentage.

[4] Percentage of injections per prescription: Percentages, calculated by dividing the number of patient encounters during which an antibiotic or injection is prescribed, by the total number of encounters surveyed, multiplied by 100.

[5] Percentage of drugs prescribed from the EML; Percentage, calculated by dividing the number of products prescribed which are listed on the essential drugs list or local formulary (or which are equivalent to drugs on the list) by the total number of products prescribed, multiplied by 100.

## Results:

The data were entered in Microsoft Excel 2010 and analysed using SPSS 22 software for frequency distributions and percentages to assess the prescribing indicators. A total of 50 prescriptions were analysed over one month. The demographic distribution of patients mirrored a rising trend with increasing age as the higher proportion of patients were 30-50 years of age. Both males and females were almost equal in proportion. There were multiple and diverse diagnoses. Hence, we categorized it into communicable and non-communicable diseases and the majority had non-communicable diseases (Table 5 - see PDF). It was found that a total number of 301 drug products had been prescribed in the 50 patient encounters and thus, the average number of drugs per prescription was 3.48% and the standard deviation was 1.32. Moreover, the median number of drugs per prescription was 4. Overall, the study revealed a higher value for this indicator than the standard reference (Table 1 - see PDF). It was found that 13.79% of prescriptions have generic names, whereas 27.58% of patient encounters had at least one drug from the national list of essential medicines list (NLEM 2015). It was evident that 6.08% of prescriptions have antibiotics and around 0.67% have been prescribed as injections.

The number of prescriptions with fixed drug combinations was 27.55%. Among the prescriptions analysed for FDCs composition, it was found that the total number of FDCs having 2 drugs was 53%, the three-drug combination was 14%, the four-drug combination was 4%, five drug combination was 8% and more than 5 drugs combinations was 3% (Table 3 - see PDF). The most common one being prescribed was metformin plus glimepiride for type 2 diabetes followed by pantoprazole plus domperidone for gastritis. Prescriptions analyzed for the number of drugs per prescription showed that patient encounters with two drugs were (18%), three drugs (20%) and four drugs (20%) accounting for a total of prescriptions falling under either of these three categories, 14% with 5 drugs and 4% with six drugs and 14% with seven drugs respectively (Table 4 - see PDF). The most highly prescribed antimicrobial agent was amoxicillin–clavulanic acid, anti-helminthic was albendazole, anti-fungal was itraconazole followed by ketoconazole. The most commonly prescribed anti-malarial drugs combination was the artemether-lumefantrine combination, which was not approved for use in central India, where artesunate plus sulfadoxine-pyrimethamine are recommended [9]. The most common indication for antibiotic use was found to be a variety of respiratory and urinary tract infections. The most common drug prescribed for acute urinary tract infection was nitrofurantoin (Table 2 - see PDF).

## Discussion:

Core drug prescribing indicators measure the prescribing practices and performance of healthcare providers concerning the rational use of medicines. The core prescribing indicators for the prescriptions in the department of internal medicine were assessed in the study institute based on a sample of 50 patient encounters that took place at the OPD in the dept. of Internal medicine. The data that were collected prospectively by analysing the prescriptions demonstrated that the average number of drugs prescribed per encounter was 3.48%. Comparison to the standard range advocated by the WHO for this indicator which estimates the degree of polypharmacy revealed that the measured average was much higher than the reference range of 1.6-1.8 which was considered ideal [10]. The same was seen in FDC drugs, where a high percentage of fixed drug combinations were prescribed in addition to the use of a combination of different drugs for a single indication in one patient encounter. Some of the Indian studies which were conducted using the WHO core prescribing indicators have shown similar results which were unlike our results, where they had mentioned 2.955%, 3.76 % and 4.98% respectively [11- 13]. The high average number of drug products per prescription exceeding the WHO reference range demonstrates that a high degree of polypharmacy is prevalent in our centre which might be due to the high prevalence of non-communicable metabolic diseases such as hypertension, diabetes, and coronary vascular diseases and dyslipidaemia which are often coexistent contributing to the need for management of more than one disease entity in a single patient simultaneously (14). India is a major country suffering from the burden of diabetes globally. The prevalence of diabetes in adults aged 20 years or older in India increased from 5·5% in 1990 to 7·7% in 2016(15). In our study, we encountered the same fact that a high proportion of prescriptions had the diagnosis of non-communicable disease with diabetes ranking highest. In this type of patient prescribing FDCs is a rationale, due to the increasing requirement of drugs in patients with more than one disease [16-17]. But recently unfortunately 424 FDCs were banned due to inappropriate combinations, so prescribers should be vigilant about prescribing rationally in FDCs. In our study, the number of prescriptions with fixed drug combinations was accounting for 27.55%. In a similar study, they were encountered to be 32.57% [11]. In the present study, only 27.58% of prescriptions were from the current list of essential medicines (NLEM 2015). This could be due to the lack of sensitization of the physicians and the lack of rules being enforced to mandate prescribing from the essential drugs list. Around 6.08% of prescriptions have antibiotics and a very less percentage (0.7%) have been prescribed as injections, whereas the recommended range by WHO was 13.4-24.1%, which will show the rationale for prescribing antibiotics and injections in the current study centre. In the present study, only 13.79% prescribed medicines with a generic name. Previous studies in a tertiary care teaching hospital found that almost 100% of prescriptions were with generic names. Other studies of the western part of India had similar observations to our study where only 0.05 % of the drugs out of 1842 products were prescribed in the generic name [11,18].

Results of a spate of similar studies have shown that the higher the doctor's education and training experience, the proportion of drugs they prescribed by generic names showed a decline, and attitudinal differences have been seen in physicians in low- and middle-income countries compared to those in high-income countries [19-20]. Hence, frequent clinical prescription audits along with training on good prescribing practices to clinicians improve the quality of prescribing practice [21-22]. The most common reason for the low percentage of generic prescribing could be due to repeated and effective promotion of the branded products by pharmaceutical companies and in certain instances, clinicians are forced to agree to the insistence of patients demanding the latest medicines for treatment, and the presumed belief among a subset of prescribing physicians that the quality differences between generic and brand drugs could adversely affect the therapeutic outcomes. Such an opinion could affect the prescribing practice of generic drugs and leads to confusion among people. Sometimes the pharmaceutical industries play an important role in branded drug prescription, by offering financial aid to prescribers like free foreign visits. Previous studies have also shown that prescribing with the generic name was more in public centres in comparison to that in private sector hospitals [23]. We have to increase awareness of generic prescribing, considering the burden due to the high cost of treatment on the public by the practice of brand name prescribing. Another study on the cost differences in prescribing generic vs. brand name prescribing in chronic disease patients concluded that all generics were more than 40% cheaper, per defined daily dose per month than the brand version [24]. In low-economic countries, generic prescribing is much more helpful to the public. This practice can be increased by an integrated approach of training the medical students who are future prescribers about the pharmacoeconomic significance in their routine pharmacology study course, in addition to conducting regular continuous medical education programs (CME) for clinicians with the focus of alleviation of their doubts on quality or bioequivalence regarding the use of generic medicines. Governments should also ensure quality control of generic medicine as a part of an ongoing exercise, routinely conducted by the US FDA. A variety of strategies have been recommended by experts to overcome the barriers to genetic prescribing and the most vital of these include enforcing statutory obligations, setting clear guidelines for generic prescribing and legally de-incentivizing prescribing by propriety name [25-29]. A major limitation of our study is the number of prescriptions. This study implies the need for implementing interventions such as continuous medical education programs and workshops to improve awareness of rational prescribing among the medical fraternity. As our study has been conducted in a government institute of national importance, the pitfalls that we found in our prescription practices should be improved for the benefit of the public.

## Conclusions:

With this, we conclude that our study of the prescribing patterns of drug use by using WHO core prescribing indicators has clearly shown the prescribing practices for essential medicine list, fixed drug combinations and generic prescribing were injudicious and irrational. A regular trend of poly pharmacy was found and inappropriate use of multivitamins was seen. Therefore, we conclude that frequent prescription audit studies provide a bridge between areas like rational use of drugs, pharmaco economics, evidence-based medicine, pharmaco genomics and pharmaco vigilance. Hence, we suggest appropriate measures like teaching pharmaco economics, rational prescribing to medical students during their undergraduate level only, regular CMEs and training workshops for clinicians on these two issues particularly on generic prescribing should be implemented by policymakers and administrators to reduce prescribing with a brand name, irrational fixed drug formulations, and injudicious multivitamin prescription. Encouraging clinicians to practice the prescribing of medicines from the list of essential medicines is a must. The administrative team and policymakers should implement all these essential needs to ensure rational and safe prescribing.
